# Insight into Differential Responses of Upland and Paddy Rice to Drought Stress by Comparative Expression Profiling Analysis

**DOI:** 10.3390/ijms14035214

**Published:** 2013-03-04

**Authors:** Xipeng Ding, Xiaokai Li, Lizhong Xiong

**Affiliations:** 1 National Key Laboratory of Crop Genetic Improvement and National Center of Plant Gene Research (Wuhan), Huazhong Agricultural University, Wuhan 430070, China; E-Mails: dingxipeng@webmail.hzau.edu.cn (X.D.); lixiaokai1989726@webmail.hzau.edu.cn (X.L.); 2 Tropical Crops Genetic Resources Institute, Chinese Academy of Tropical Agricultural Sciences, Key Laboratory of Crop Gene Resources and Germplasm Enhancement in Southern China, Ministry of Agriculture, Danzhou, Hainan 571737, China

**Keywords:** *Oryza sativa*, expression pattern, drought resistance, quantitative trail loci, near isogenic lines

## Abstract

In this study, the drought responses of two genotypes, IRAT109 and Zhenshan 97 (ZS97), representing upland and paddy rice, respectively, were systematically compared at the morphological, physiological and transcriptional levels. IRAT109 has better performance in traits related to drought avoidance, such as leaf rolling, root volumes, the ratio of leaf water loss and relative conductivity. At the transcriptional level, more genes were induced by drought in IRAT109 at the early drought stage, but more genes had dynamic expression patterns in ZS97 at different drought degrees. Under drought conditions, more genes related to reproductive development and establishment of localization were repressed in IRAT109, but more genes involved in degradation of cellular components were induced in ZS97. By checking the expression patterns of 36 drought-responsive genes (located in 14 quantitative trail loci [QTL] intervals) in ZS97, IRAT109 and near isogenic lines (NILs) of the QTL intervals, we found that more than half of these genes had their expression patterns or expression levels changed in the NILs when compared to that in ZS97 or IRAT109. Our results may provide valuable information for dissecting the genetic bases of traits related to drought resistance, as well as for narrowing the candidate genes for the traits.

## 1. Introduction

Drought stress is one of the major environmental factors limiting rice (*Oryza sativa* L.) production. Drought can occur at any stage during the rice growing season due to inadequate irrigation, uneven distribution of rainfall, variation in the rainfall patterns from one year to another or inadequate rainfall in large areas [1]. The reproductive stage (from anthesis to flowering) is recognized as the most critical stage at which drought stress can cause harm to the crop [2,3].

The study of the genetic and molecular bases of drought resistance has always been challenging, because drought resistance of crops consists of many complex traits reflected in morphological and physiological characteristics, and different mechanisms often combine to confer drought resistance. The mechanisms of drought resistance include drought escape, drought avoidance, drought tolerance and drought recovery, among which drought avoidance and drought tolerance are the two major mechanisms for drought resistance in rice [4,5]. Drought avoidance helps plants maintain good water potential by enhancing water uptake and reducing water loss (e.g., an effective rooting system, leaf rolling and fast stomatal closure). The ability to maintain cellular turgor under dehydration stress via osmotic adjustment and antioxidant capacity is considered an important drought tolerance mechanism. Drought recovery is an important mechanism when drought occurs early in rice development. Under different environmental conditions and at different developmental stages, the mechanisms of drought resistance differ. For example, at the reproductive stage in rice, drought avoidance was a more important genetic basis of drought resistance than drought tolerance in sandy soil, whereas drought tolerance may play a more important role in the genetic control of drought resistance in paddy soil [6].

As a powerful high-throughput tool, cDNA microarrays (*i.e.*, gene chip technology) have been widely used to detect gene expression profiles in many model organisms, including Arabidopsis and rice, and under many abiotic stress conditions [7–12]. To understand the genetic mechanism of drought resistance in rice, expression patterns were detected at different durations and degrees of drought stress in various cultivars [12–15]. Upon polyethylene glycol treatment, genes that may play a role in detoxification, protection against oxidative stress and maintaining cellular turgor have higher expression in upland rice, but genes involved in the degradation of cellular components have higher expression in lowland or paddy rice [12]. Drought stress induced more genes related to degradation processes in the sensitive genotypes than in the tolerant genotypes, whereas more genes related to water use and photosynthesis were downregulated in the tolerant genotypes than in the sensitive genotypes [13]. Transcriptomic analysis of a drought-tolerant rice Nagina 22 *versus* a drought-susceptible rice, IR64, was reported by Wang [14]. Several enzyme-encoding genes were induced in Nagina 22 (N22), and regulatory components that confer drought tolerance were repressed in IR64 under drought stress. Significant upregulation of the α-linolenic acid metabolic pathway was observed in N22 under drought. Genome-wide temporal-spatial gene expression profiling of drought responsiveness revealed that most of the differentially expressed genes were under temporal and spatial regulation, suggesting a crosstalk between various development cues and environmental stimuli [15]. However, none of the reported comparative profiling analyses were performed in association with a population that has been investigated for genetic control of drought resistance.

An inbred line population derived from a cross of paddy rice Zhenshan 97 (ZS97) and upland rice, IRAT109, has been intensively investigated for the genetic control of drought resistance [8,16,17], and near isogenic lines (NILs) for many quantitative trait loci (QTL) related to drought resistance have also been constructed in this population [18]. In this study, the expression profiles of the two parents, ZS97 and IRAT109, under normal and drought conditions at the reproductive stage were investigated by the Affymetrix GeneChip. More genes related to reproductive development and establishment of localization were downregulated by drought in IRAT109. The expression levels of genes involved in degradation of cellular components were higher in ZS97 than in IRAT109 under drought conditions. More genes were induced by drought in IRAT109 at a second time point, and more genes had dynamic expression patterns in ZS97 at different degrees of drought treatment. In this study, we further investigated the expression patterns of dozens of drought-responsive genes in the isogenic background.

## 2. Results and Discussion

### 2.1. Performance of Upland and Paddy Rice under Normal and Drought Conditions

**Figure 1:**
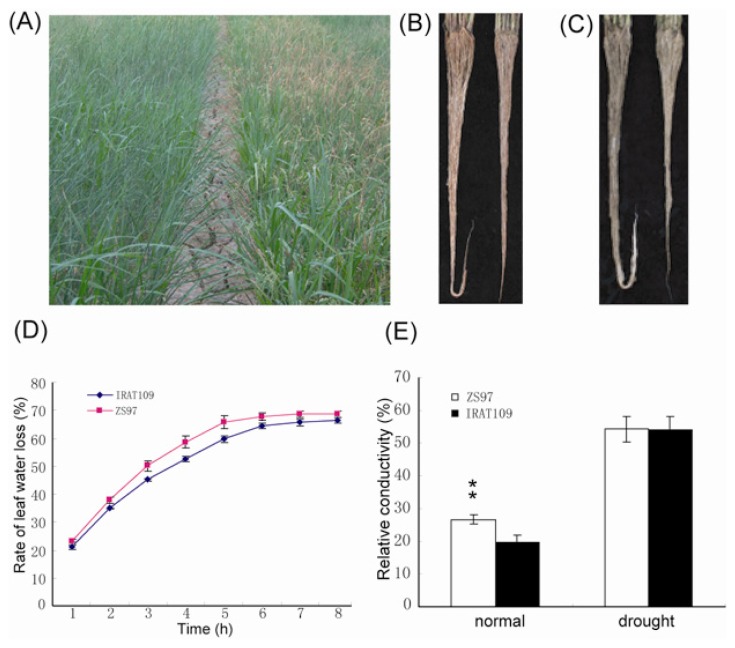
The performance of ZS97 and IRAT109 under normal and drought conditions. (**A**) Performance of ZS97 and IRAT109 under drought stress. Left: IRAT109, right: ZS97; (**B**,**C**) Root under normal and drought conditions, respectively. Left: IRAT109, right: ZS97; (**D**) Rate of leaf water loss of ZS97 and IRAT109; (**E**) Relative conductivity of leaves from ZS97 and IRAT109 under normal and drought conditions.

Upland rice has been widely recognized to be more drought resistant than paddy rice. Previously, we conducted a serious genetic analyses of drought resistance using a recombined inbred line (RIL) population derived from the paddy genotype, ZS97, and upland genotype, IRAT109 [16,17]. To further investigate the difference of the two genotypes in response to drought in association with differential gene expression (described next), we compared the morphological and physiological responses and yield-related traits of the genotypes under both irrigated and drought-stressed cultivation. Under normal conditions, ZS97 showed significantly higher trait values than IRAT109 in grain yield (GY), tillers per plant (TPP) and spikelet fertility (SF), but IRAT109 showed significantly greater plant height (PH), number of spikelets per panicle (SPP), 1,000-grain weight (KGW), total root volumes (RV) and root volumes per tiller (RVT) (Table 1, Figure 1B). The ZS97 leaves lost water significantly faster (*p* < 0.01) than IRAT109 leaves (Figure 1D). The relative electrical conductivity of leaves (REC) of ZS97 leaves was significantly higher (*p* < 0.01) than that of IRAT109 (Figure 1E). Under drought-stressed cultivation, ZS97 had significantly greater TPP and SPP, but lower GY, PH, KGW, RV and RVT than IRAT109 (Figure 1D), while the value of SF indicated no significant difference. The leaves of IRAT109 rolled faster than those of ZS97 under drought stress at the reproductive stage. In fact, leaves of ZS97 seldom rolled, but became dry at the later stage of drought stress (Figure 1A). After re-watering, the degree of leaf drying of ZS97 was more severe than that of IRAT109. The relative values of the above traits have been widely used as indices to evaluate drought resistance [19,20]. We noted that the relative values of GY, SF, KGW and RVT were significantly higher in IRAT109 than in ZS97, but the relative values of TPP and SPP were lower in IRAT109 than in ZS97 (Table 1). These results suggest that IRAT109 performs better (in terms of grain yield) than ZS97 only under drought stress conditions, which may be mainly due to the better performance of IRAT109 in traits related to drought resistance, such as leaf rolling and root volumes.

### 2.2. Different Drought-Responsive Profiles of the Two Rice Genotypes

Because the two rice genotypes showed distinct responses to drought stresses, we further analyzed the whole genome expression profiles of the two genotypes under normal cultivation and different degrees of drought stress conditions. Drought stress was monitored based on morphological phenotypes (leaf-rolling status) and relative water content (RWC) of leaves. To assess the quality of the gene chip experiment, three preliminary tests were conducted. First, 2000 genes with the highest expression coefficient of variation among all genes in the 16 arrays were selected and used to analyze the correlation coefficient between biological replicates. All eight correlation coefficients between biological replicates were greater than 0.99, indicating high quality of the sample preparation and gene chip hybridization (Figure S1). Second, we checked the expression levels of many previously reported drought-responsive genes, including *OsBURP03*, which encodes an RD22-like protein and is induced by drought stress [21]. All of them were upregulated in this experiment, suggesting that the drought treatment was successful. Finally, we confirmed the gene chip data byreal-time PCR analysis of 36 genes. Except for minor differences for three genes, the results obtained by the two methods were highly consistent for the other 33 genes (Figure S1). Considering that the primers for real-time PCR are gene specific, the variation of three genes generated by the two methods was likely due to cross-hybridization in the gene chip experiment. Collectively, these results indicated that the microarray experimental data were of high quality.

**Figure 2:**
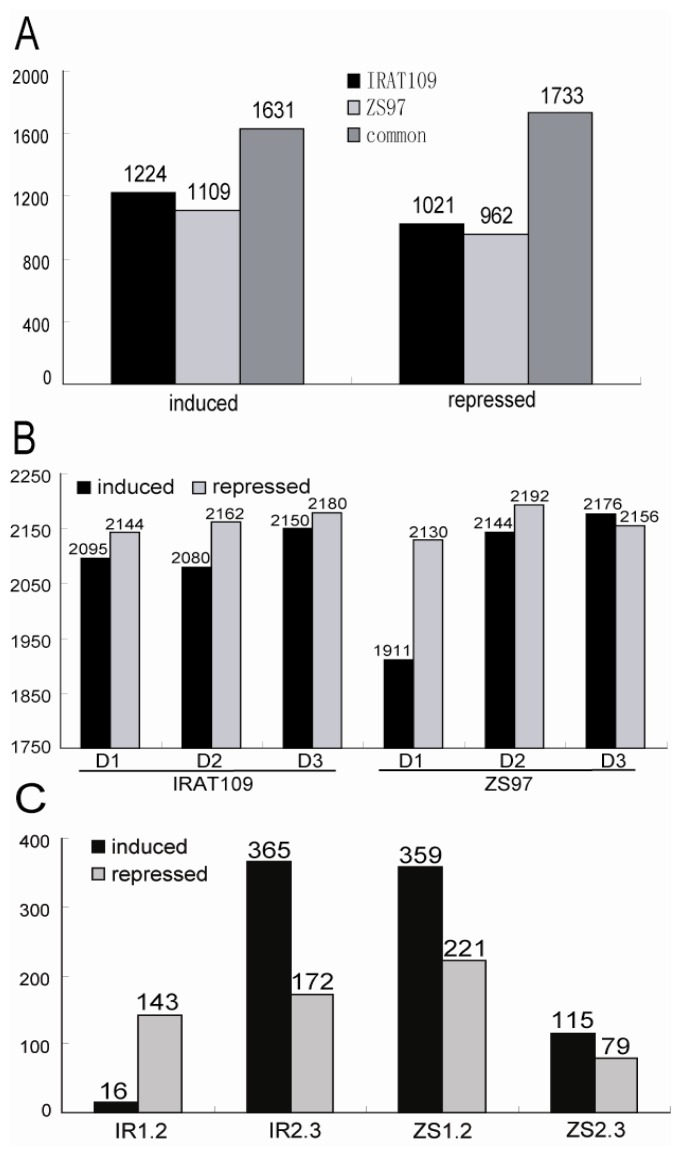
The number of differentially expressed genes (with *p* < 0.05 as threshold). (**A**) Total number of induced and repressed genes in IRAT109, ZS97 and both; (**B**) Numbers of genes significantly up- or downregulated at D1, D2 and D3 stages of drought stress compared to non-stress condition; (**C**) Number of differentially expressed genes among different time points. IR1.2 and ZS1.2: number of differentially expressed (*i.e.*, significantly up- or downregulated) genes between D1 and D2 in IRAT109 and ZS97, respectively. IR2.3 and ZS2.3: number of differentially expressed genes between D2 and D3 in IRAT109 and ZS97, respectively.

We first compared the total number of drought-responsive genes between the two genotypes. In IRAT109, 2855 and 2754 genes were upregulated and downregulated, respectively, and in ZS97, 2740 and 2695 genes were upregulated and downregulated by the drought stress. Although most of the drought-responsive genes were the same in the two genotypes, a significant proportion of the genes showed differential responses between the two genotypes. There were 1224 and 1021 genes that were upregulated and downregulated, respectively, only in IRAT109, and 1109 and 962 genes that were upregulated and downregulated only in ZS97. There were 1630 and 1733 genes that showed induced and repressed expression, respectively, in both genotypes (Figure 2A).

We then compared the responsive genes in the three samples with different degrees of drought stress (D1 to D3). The numbers of upregulated genes were 2,095, 2,080 and 2,150 in IRAT109 at D1, D2 and D3, respectively, and 1,911, 2,144 and 2,176 in ZS97. The numbers of downregulated genes were 2144, 2162 and 2180 in IRAT109 at D1, D2 and D3 and 2130, 2192 and 2,156 in ZS97. There were 1470 and 1,441 genes upregulated and 1,627 and 1,644 genes downregulated at all three stress stages in IRAT109 and ZS97, respectively (Figure 2B). The number of upregulated genes was obviously higher in IRAT109 than in ZS97 under slight drought conditions (D1). The number of genes showing significantly different expression changes between D1 and D2 was higher in ZS97 (580 genes) than in IRAT109 (159 genes). This result implies that the upland genotype may respond to early drought stress more efficiently than the paddy genotype by upregulating more stress-related genes. In addition, the number of genes showing significantly different expression changes between D2 and D3 was lower in ZS97 (194 genes) than in IRAT109 (537 genes), which implies that the upland rice may have more genes that maintain strong induction or more genes that are induced at the severe stress stage (Figure 2C).

GO analysis was performed for upregulated and downregulated genes (Table 2). The results indicated that genes related to stress, external stimuli, embryonic development and the carbohydrate metabolic process were upregulated, whereas genes related to photosynthesis, cell differentiation, the phosphorus metabolic process and the reproductive process were downregulated by drought in both ZS97 and IRAT109. For example, nine of 12 genes related to the response to water stress were induced, and nine of 16 genes related to photosynthesis (light-harvesting) were repressed by drought stress in both ZS97 and IRAT109. More genes related to the reproductive process were downregulated by drought in IRAT109 than in ZS97. According to the predicted function of the genes, we speculated that some of these genes may be related to the significantly decreased SPP under drought stress in IRAT109. Interestingly, some genes involved in the degradation of cellular components (e.g., secondary compound metabolic process and nitrogen compound catabolic process) were induced significantly only in ZS97, and some genes categorized in the GO analysis as cell cycle process and transcription were induced significantly only in IRAT109. In addition, some genes related to transport were repressed by drought only in IRAT109. The expression levels of more genes with hydrolase activity and oxidoreductase activity were upregulated in ZS97. More genes with nutrient reservoir activity and nucleic acid binding function were induced, and more genes with hydrolase activity were repressed in IRAT109. Such differences in the number of responsive genes categorized under different GO terms further indicate a potential link between the differential drought responses of the two genotypes and the molecular basis at transcriptional level.

### 2.3. Dynamic Patterns of the Drought-Responsive Genes

**Figure 3:**
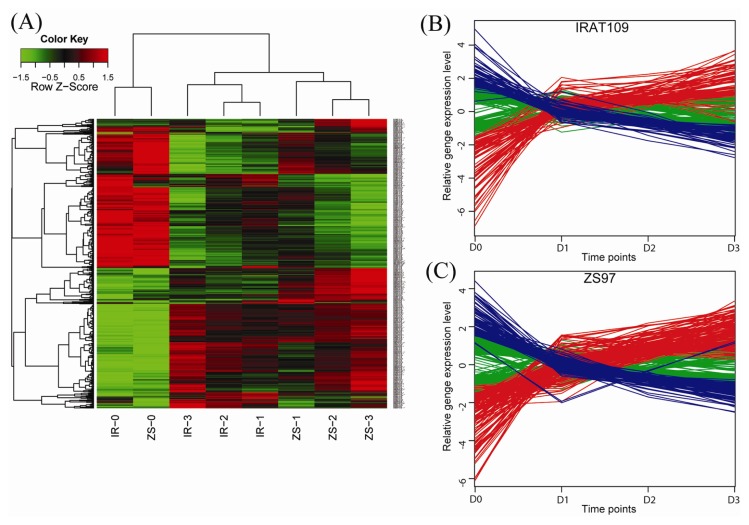
Differentially expressed genes during the time course of drought (D0 to D3). A total of 925 genes (625 in ZS97 and 320 in IRAT109) were differentially expressed among the four stages of drought stress. (**A**) Hierarchical clustering of differentially expressed genes based on the correlation coefficients of the relative gene expression values (Z-score) for each time point. Red and green indicate high and low expression levels; (**B**, **C**) Two groups of genes with opposite expression trends from D0 to D3 in IRAT109 and ZS97, respectively. The normalized expression levels (based on the ln-transformed mean of the four stages) of each gene are illustrated. The colored lines denote the expression profile of the genes: blue indicates a large decrease from D0 to D3 and red indicates a large increase and green indicates a small change. Stages: D0, before drought stress; D1, D2 and D3: three stages of drought stress.

We further investigated the dynamic expression changes of the drought-responsive genes during the drought stress development (*i.e.*, from D0 to D3). In IRAT109, 320 drought-responsive genes showed dynamic expression changes (Figure 3). Among them, 170 genes showed gradually decreased expression, whereas 150 genes showed increased expression from D0 to D3. In ZS97, 605 drought-responsive genes showed dynamic expression changes during the drought stress development. Among them, the expression levels of 280 genes were gradually decreased, and 325 genes were increased from D0 to D3. Those genes with increased expression levels included many genes related to improve drought resistance, such as *OsLEA3-1*. The relative yield of rice plants overexpressing *OsLEA3-1* was significant higher than that of wild-type rice plants [22]. The majority of the genes with gradually increased expression were attributed to the GO terms response to water stress in ZS97 and RNA biosynthetic and metabolic processes in IRAT109. The expression levels of most genes related to transport and transmembrane transport were gradually decreased in ZS97 during the drought stress.

In our dataset, the expression levels of about 90% of genes were stable in both IRAT109 and ZS97 at different degrees of drought stress, including the 19 genes that have been proven to have uniform expression based on the microarray data of 31 tissues or organs [23]. To further investigate the stability of the 19 genes and five internal control genes (*i.e.*, actin-1, ubiquitin fusion protein, elongation factor1-alpha, tubulin beta-6 chain and GAPDH) referred to in many reports [7,24] under different abiotic stresses, we checked them in the published microarray datasets [25] and several unpublished datasets for different abiotic stress treatments, including cold for different varieties, heat and drought. The coefficient of variation of all five internal control genes was higher than 20%. Among the 19 suggested constitutive genes, nine showed highly stable expression under different abiotic stress conditions and their coefficient of variation was less than 20% (Table S2). These results provided more options for selecting internal control genes in quantitative expression analyses of genes under drought stress in rice.

### 2.4. Differentially Expressed Genes in the Intervals of Drought Resistance-Related QTL

Because genetic analysis of drought resistance at the reproductive stage has been conducted in the RIL population derived from the two genotypes ZS97 and IRAT109 [8,17], we further investigated the genomic distribution of the drought-responsive genes and their locations associated with the drought-resistance QTL detected in the population. There were a total of 4,316 genes differentially expressed between ZS97 and IRAT109 under drought stress. These genes were used for genomic distribution analysis basing on the TIGR 6.1. In the genomic regions of 17 QTL, the number of all ORFs, containing responsive and no-responsive under drought stress, in one QTL interval ranged from 139 to 653 (Table S3). However, only 213 differentially expressed genes were located in the 17 QTL intervals, and 92 of them were upregulated and 121 of them were downregulated by drought stress (Table S4). The number of drought-responsive genes ranged from 3 to 28 in each QTL interval (Table S3).

To investigate the function of these 213 differentially expressed genes located in the intervals of the 17 QTL, GO analysis was performed. The result indicated the enrichment of genes involved in biological processes (*i.e.*, response to stimuli [response to endogenous stimulus and response to biotic stimulus], growth [flower development] and cell communication [signal transduction]) and genes involved in molecular functions (*i.e.*, nucleotide binding [purine nucleoside binding, adenyl nucleotide binding and ribonucleotide binding], carbohydrate binding, kinase activity [transferase activity, protein tyrosine kinase activity and protein serine/threonine kinase activity]). Protein kinases are involved in many biological processes, including responses to stress. Among these 213 genes, 25 are predicted to be putative kinases, including receptor kinases and two wall-associated kinases (OsWAK29 and OsWAK10d). For example, OsSIK1, a recently identified receptor-like kinase that confers drought and salt tolerance in rice through activation of the antioxidative system [26], showed upregulation in the upland rice IRAT109. They may play an important role in receiving and transducing exoteric drought signals and the regulation of expression level or activity of downstream genes.

### 2.5. Differential Expression Patterns of Drought-Responsive Genes in the QTL Intervals

To verify the differential gene expression patterns, 36 drought-responsive genes located in 14 QTL intervals were selected for real-time PCR analysis using the same stressed samples of the two genotypes as used for the gene chip analysis (Table S1). This analysis also served as a quality check of the gene chip experiment, as described above. The number of selected genes distributed in each QTL interval ranged from one to three, and all of them showed differential expression between ZS97 and IRAT109, based on the gene chip results (Table S1). We also included 14 pairs of NILs of the QTL in the real-time PCR analysis to compare the gene expression patterns between the parental genotypes and the NILs. Each pair of NILs refers to the ZS97 (NIL-ZS) and IRAT109 (NIL-IR) genotypes for the target QTL interval. The 14 pairs of NILs showed a significant difference in at least one trait under normal or drought stress conditions [18].

**Figure 4:**
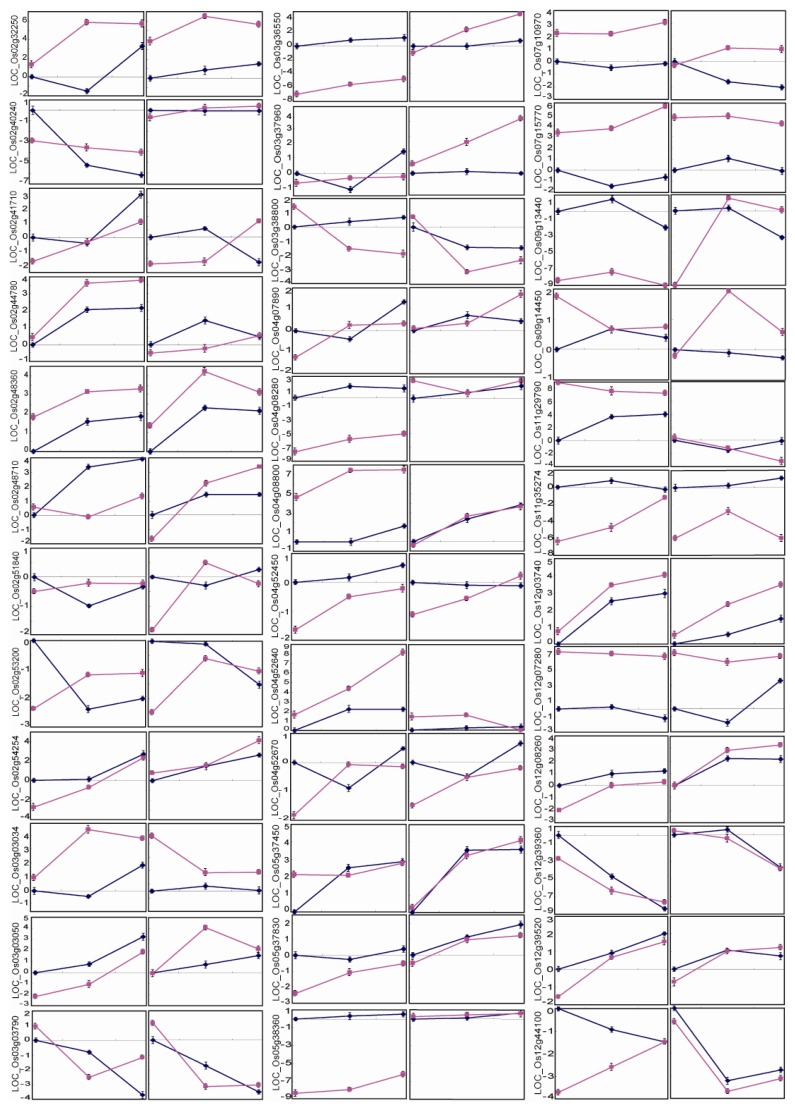
Real-time PCR analysis for 36 genes in ZS97, IRAT109, NIL-ZS and NIL-IR. The left figure for each gene illustrates its expression profile in ZS97 and IRAT109; right figure illustrates its expression profile in NIL-ZS and NIL-IR. The colored lines denote the expression profile of the 36 drought-responsive genes: blue indicates allele from ZS97 and red indicates allele from IRAT109. The x-axes are three time points of drought stress treatment, and the y-axes are scales of transcription ratios determined from the real-time PCR were ln-transformed. D0, before drought stress; D1 and D2, two stages of drought stress.

Under normal conditions (D0), 29 of the 36 genes were differently expressed between the two parents, whereas 17 of the 29 genes had equivalent expression level between the paired NILs corresponding to the QTL intervals in which the analyzed genes were located (Figure 4, Table 3). The expression level of one gene (*LOC_Os04g08280*) was higher in ZS97 than in IRAT109, but lower in the NIL-ZS than in NIL-IR. Among the seven genes with equivalent expression levels between the two parents, three genes were differently expressed between the corresponding NIL pairs. These results suggest that more than half of the analyzed genes had different expression patterns in the NIL background compared to the patterns in the two parents under normal growth conditions.

Under drought stress conditions, the 36 genes can be divided into four groups based on differences in the drought-responsive trend between the parent line and the corresponding NIL (Figure 4, Table 3). In the first group (eight genes), the responsive trend was different both between ZS97 and NIL-ZS and between IRA109 and NIL-IR. In the second group (seven genes), the responsive trend of the ZS97 allele differed between ZS97 and NIL-ZS, but the responsive trend of the IRAT109 allele was the same between IRAT109 and NIL-IR. In the third group (seven genes), the case was opposite that in the second group (same responsive trend between ZS97 and NIL-ZS, but different between IRAT109 and NIL-IR). The fourth group included 14 genes. Among them, 11 genes showed similar responsive trends to drought between both the parents and between the paired NILs; two genes (*LOC_Os03g03790* and *LOC_Os12g39360*) were downregulated and nine genes were upregulated by drought. However, the relative expression levels of these genes were significantly different under normal conditions or their fold-change values under drought stress were significantly different between the parents or between the paired NILs. The other three genes of the fourth group showed a similar trend between each parent and the corresponding NIL, but their responsive patterns were reversed between the parents or between the paired NILs. Among them, one gene (*LOC_Os02g53200*) was downregulated in ZS97 and NIL-ZS, but was upregulated in IRAT109 and NIL-IR by drought, and two genes had no significant change in ZS97 and NIL-ZS, but were induced after drought stress. These results suggested that most of the analyzed genes had a different drought-responsive trend or amplitude in the NIL background compared to the parent, even though the genotypes of the target intervals in which the analyzed genes were located are the same, suggesting that genetic background has a great effect on the expression of drought-responsive genes.

The 36 genes can be also divided into four groups based on their responsive trend to drought between the two parents and/or between the paired NILs (Figure 4, Table 3). The first group (11 genes) showed the same responsive trend both between ZS97 and IRAT109 and between NIL-ZS and NIL-IR. In the second group (eight genes), the responsive trend was different between ZS97 and IRAT109, but was the same between NIL-ZS and NIL-IR. The third group (nine genes) showed different responsive trend between NIL-ZS and NIL-IR and between ZS97 and IRAT109. The fourth group (eight genes) showed the same responsive trend between ZS97 and IRAT109, but different responsive trend between NIL-ZS and NIL-IR. These results indicated that most of the genes with different drought-responsive patterns between the parents had their responsive patterns changed in the paired NILs, further illustrating the great effect that genetic background has on the expression of drought-responsive genes.

### 2.6. Discussion

#### 2.6.1. Mechanism of Drought Resistance at the Reproductive Stage

The relative GY was higher in IRAT109 than in ZS97, which may lead to a conclusion that IRAT109 is more drought-resistant than ZS97. However, both ZS97 and IRAT109 have drought-responsive traits. In IRAT109, the detached leaves had a significantly lower rate of water loss and REC compared to those of ZS97. The IRAT109 leaves rolled faster upon drought stress, which may allow less water loss at the early stage of drought stress. These results indicate that IRAT109 has stronger water-retention ability than ZS97. In addition, the RVT was higher in IRAT109 than ZS97 under both normal and drought condition, which implies that much more water potentially can be taken up by IRAT109 than by ZS97. The relative value of SPP was lower, but RVT and biomass values [17] were higher in IRAT109 than in ZS97. In the gene chip analysis, we noted that more genes involved in the reproductive process were repressed, but more genes involved in the cell cycle process and transcription were induced in IRAT109 than in ZS97. Therefore, IRAT109 may retain higher vegetative growth than ZS97 by repressing reproductive growth (such as panicle development) to defend against drought stress. Although the REC of ZS97 leaves showed no significant difference to that of IRAT109 under drought conditions, the variation of REC before and after drought stress was smaller in ZS97 than in IRAT109. Related to this, there were significantly fewer drought-responsive genes involved in transport and transmembrane transport in ZS97 than in IRAT109, and these genes were repressed later and more slowly in ZS97 than in IRAT109. This result suggested the maintenance of ion balance may be more important to drought resistance in ZS97.

All correlation coefficients among different degrees of drought treatment were more than 0.92 in both ZS97 and IRAT109. Few genes showed differential expression at the four degrees of drought treatment: 605 genes in ZS97 and 320 in IRAT109. More than 90% of the genes responsive to drought were stable at different time points (D1, D2 and D3). A similar result was found in MH63, an indica paddy rice variety [27]. In the panicle of MH63, the number of genes changed by drought did not vary at different time points. Thus, at the reproductive stage, most responsive genes were changed by drought stress and the defense reaction to drought may start around time point D1. However, in the shoot of MH63, the number of genes changed by drought gradually increased from D1 to D3. This may indicate that the molecular mechanism of drought resistance is different at various developmental stages. In ZS97, about 200 fewer genes were induced at D1 than at the other two time points. Similarly, in MH63, the number of induced genes in flag leaves was lower at D1 and D2 than at D3. This result implies that the upland genotype may respond to the early stress more efficiently than the paddy genotype by upregulating more stress-related genes at the reproductive stage.

Comparative profiling showed that IRAT109 has more genes responsive to drought stress than ZS97. In particular, more genes predicted to have nucleic acid binding functions were responsive to drought and some genes involved in the cell cycle process and transcription were induced only in IRAT109. Genes with a nucleic acid binding function may be regulators for downstream genes and an expression level change may lead to more genes being responsive to drought stress. Genes involved in the cell cycle process and transcription may play an important role in maintaining active growth of the plant, for example, more roots in IRAT109, to obtain more water from soil. On the other hand, more genes predicted for hydrolase or oxidoreductase activity, and more genes involved in the degradation of cellular components were drought responsive in ZS97. Upregulation of genes for hydrolase and degradation of cellular components may increase the content of soluble substances, thus increasing the osmotic adjustment capacity. Induction of genes for oxidoreductases may improve drought tolerance of ZS97 plants through the antioxidation system. The comparisons of drought resistance and expression profiles between the two parents suggest that drought tolerance and drought avoidance (root traits and water-retention ability) may be the major drought-resistance mechanisms in paddy rice ZS97 and upland rice IRAT109, respectively.

#### 2.6.2. Candidate Genes for QTL Related to Drought Resistance

Plants alter their gene expression pattern in response to diverse abiotic stresses, including drought. These transcriptional changes are considered to be a major regulatory mechanism for plants to respond to abiotic stresses. Comparison of transcriptional changes in response to drought between sensitive and resistant varieties, to deduce possible candidates for QTL related to drought resistance, has been attempted in rice [13,28]. Hazen [28] identified several candidate genes for five QTL related to osmotic adjustment by investigating differential expression patterns in materials that differ in osmotic adjustment and their genomic location by matching with known QTL.

The expression level of eukaryotic genes is regulated by both *cis*-elements and *trans*-regulatory factors. Therefore, the expression patterns of candidate genes that differ between two genetically diversified parents may not maintain the same pattern in a NIL background. To test this, we checked the expression patterns of 36 drought-responsive genes in 14 QTL intervals in both the parents (ZS97 and IRAT109) and the paired NILs (NIL-ZS and NIL-IR). Surprisingly, 17 genes had significantly different responsive strength to drought stress between ZS97 and NIL-ZS. The responsive trends of three genes, *LOC_Os02g41710*, *LOC_Os07g15770* and *LOC_Os11g29790*, were opposite between ZS97 and NIL-ZS. Similarly, 15 genes had different drought-responsive trends and six genes showed opposite response trends between IRAT109 and NIL-IR. Of the 36 genes, the drought-responsive expression levels of 29 genes were significantly different between the two parents under normal conditions, but only 12 of these genes showed the same difference between NIL-ZS and NIL-IR as between the parents (Figure 4, Table 3). These results suggest that identifying candidate genes by comparing the expression profiles of parental lines with distinct genetic backgrounds should be adopted with caution. Such caution is also needed when trying to identify candidate genes by comparing the sequence polymorphism of candidate genes [29]. Of 35 possible candidate genes in the selected intervals, only six were found to be polymorphic between the parents; and of the six genes, only one was closely linked to the QTL [29].

Because the difference of genetic background has been dramatically reduced in the paired NILs, the differentially expressed genes in the target interval are most likely candidate genes (or are at least related to them) for the phenotypic difference of the target trait if they maintain the differential expression patterns between the paired NILs. For example, the expression levels of two different alleles of *GHD7* (*LOC_Os07g15770*), an important regulator of heading date, plant height and yield potential in rice [30], were significantly different between ZS97 and IRAT109, as well as between a pair of NILs (*N24-ZS* and *N24-IR*) for the interval containing *GHD7*, and this pair of NILs indeed showed a significant difference in the traits, including heading date, plant height and grain yield under both normal and drought conditions [18]. Had the function of *GHD7* not been reported, this information would be very helpful for pinpointing the candidate gene for heading date, plant height and yield trait in N24. Among the 14 pairs of NILs checked in this study, significant phenotypic differences were observed for 10 pairs of NILs under normal growth conditions [18], and at least one gene in the corresponding interval showed differential expression between NIL-ZS and NIL-IR for seven pairs of NILs under normal conditions. Except *GHD7*, 10 genes located in the seven QTL intervals showed differential expression between NIL-ZS and NIL-IR and can be considered as candidate genes for the quantitative traits for future studies.

Under drought stress conditions, 28 of the 36 genes located in the QTL intervals showed different expression levels and/or different responsive trends between NIL-ZS and NIL-IR. Although such differences between paired NILs are not the same as the differential expression between ZS97 and IRAT109 for some genes, these genes can serve as candidates for some of the drought-resistance QTL for further validation. For example, nine pairs of NILs showed significant differences in phenotypic trait values under normal and drought stress conditions. In the target intervals of the nine QTL, however, only 10 genes, distributed in six intervals, showed differential expression between NIL-ZS and NIL-IR under normal and drought stress conditions. Four NILs (N4, N8, N15 and N36) showed significant differences in traits only under drought stress; seven genes in the four intervals had different expression levels between NIL-ZS and NIL-IR. Among the seven genes, five were differentially expressed between NIL-ZS and NIL-IR under both normal and drought stress conditions. Two genes, *LOC_Os04g07890* and *LOC_Os11g29790*, located in the interval N15 and N36, respectively, were differentially expressed in NIL-ZS and NIL-IR only under drought stress conditions and may be considered as candidate genes for the QTL with high priority. The expression levels of *LOC_Os04g07890*, which encodes a structural maintenance of chromosomes (SMC) protein, were equivalent under normal conditions, but significantly different under drought stress conditions. The SMC proteins are involved in transcription, DNA repair and recombination and may be involved in the maintenance of chromosome structure under drought stress [31]. The gene *OsSIK1* (*LOC_Os11g29790*), which encoded a receptor kinase protein, may play an important role in signal transduction and regulation of downstream genes [26].

## 3. Experimental Methods

### 3.1. Plant Materials, Cultivation and Stress Treatment

Plants of two rice cultivars (IRAT109, a drought-resistant japonica variety, and ZS97, a drought-sensitive indica variety) were grown in PVC pipes. For half the ZS97 and IRAT109 plants, two drought-rehydration cycles were performed to each plant in PVC pipes at the booting stage (about 14 days before flowering). When all leaves of a stressed rice plant became completely rolled, watering was applied to the full capacity of the pipe, and the second cycle of drought stress was applied until all leaves became completely rolled again, according to the methods of Yue [17]; then watering was resumed for the rest of the lifecycle.

From the first drought-rehydration cycle, samples for expression profiling and relative water content (RWC) determination were harvested from the middle section of the blades of fully expanded green flag leaves of ZS97 and IRAT109 at the same time. The samples for expression profiling were immediately frozen in liquid nitrogen, and the samples for RWC determination were put into a 10-mL test tube to minimize evaporation and stored in the dark on ice. Four samples with RWC in the range of 94%–95% (no stress, D0), 83%–88% (slight drought in which leaves were slightly rolled, D1), 74%–78% (moderate drought in which about half of each leaf was rolled, D2) and 65%–69% (severe drought in which all leaves were completely rolled, D3) were collected for expression profiling analysis using an Affymetrix GeneChip. Two independent biological replicates were used for each of the normal and stressed samples.

Eight traits were scored in this study: six of them were traits of the aboveground part of the plants and the other two were root traits. The aboveground traits were related to fitness and productivity, including plant height (PH, in centimeters), grain yield (GY, in grams) and yield component traits and fertility. Grain yield and yield-related traits were measured for all plants under normal and drought stress conditions, including number of tillers per plant (TPP), number of spikelets per panicle (SPP), 1,000-grain weight (KGW, in grams) and spikelet fertility (SF, %). At the ripening stage, plants were harvested individually and air-dried to score the following traits: GY as the total weight of the grains from the whole plant, SPP as the total number of spikelets of the whole plant divided by the total number of panicles, SF as the number of filled grains divided by the total number of spikelets of the whole plant, KGW as GY divided by the number of filled grains, then multiplied by 1000. The two root traits, including the total root volumes (RV, in milliliters) and the root volumes per tiller (RVT, in milliliters), were measured at the seed maturity stage of the plants. RVT was measured as RV divided by the total number of tillers of the whole plant.

### 3.2. Microarray and Initial Data Analysis

Total RNA isolation, purification, labeling, hybridization and scanning were conducted by the CapitalBio Corporation (Beijing, China) according to Affymetrix standard protocols (http://www.affymetrix.com/products/arrays/specific/rice.affx) [32]. for the Affymetrix GeneChip, which contained 57,381 probe sets. The following steps were employed to investigate the expression profiling of ZS97 and IRAT109 under drought stress conditions. The 16 raw Affymetrix CEL files (2 varieties × 4 time points × 2 biological replicates) resulting from RNA hybridization were read into R platform (http://www.R-project.org) [33]. Background correction, quantile normalization and gene expression summarization were performed using the robust multiarray average method in the Bioconductor Affy package [34–37]. Differentially expressed genes were calculated using the Bioconductor RankProd package function, RP [38]. A gene was considered to be up- or down-regulated if the *p*-value of the RankProd analysis was <0.01, and the fold change of average expression was >2 (*i.e.*, log-fold change >1 for upregulated genes and log-fold change < −1 for downregulated genes). Differentially expressed genes between ZS97 and IRAT109 under drought stress were composed of genes that were up- or down-regulated only in IRAT109, but not in ZS97, and that were up- or down-regulated only in ZS97, but not in IRAT109. Basing on the data from TIGR version 6.1, these differentially expressed genes were searched for their genomic distributions, thus to find the differentially expressed genes in genomic regions of 17 QTL. *F*-test (*p* < 0.01) was employed to investigate the dynamic expression changes of the drought-responsive genes during the drought stress development. Gene ontology (GO) analyses (*p* < 0.01) were conducted using the Bioconductor topGO package [39] and following the method described by Wang [40].

### 3.3. Physiological Analysis

To measure RWC, flag leaves were sampled and weighed immediately for fresh weight (FW), then immersed in distilled water for 4 h at 26 °C. The turgid leaves were quickly blotted to remove the extra surface water and then weighed for turgid weight (TW) [41]. The turgid leaves were then oven-dried at 80 °C for 16 h. Finally, the dry weights (DW) of the leaves were weighted. The RWC was calculated as RWC (%) = (FW − DW)/(TW − DW) × 100.

The rate of leaf water loss (RLWL) was measured in excised leaves to investigate the ability to conserve leaf moisture. An excised leaf was weighed immediately after sampling for FW, allowed to dry under normal conditions and weighed at 1-h intervals until the weight of the dry leaf become stable. The rate of leaf water loss was calculated as RLWL (%) = (FW − weight at a designated time after being exposed to air)/FW × 100.

The electrical conductivity of leaves was measured with a conductivity meter (DDSJ-308A), according to the manufacturer’s instruction. The leaves were sampled and flushed with double distilled water, divided into approximately 0.5-cm strips, then immersed in 50 mL double distilled water at 26 °C in a shaker. After 6 h, the electrical conductivity of the liquid was measured as R1. The liquid was placed in a test tube and heated in boiling water for 15 min and then cooled to room temperature. The electrical conductivity (R2) was measured, and the relative electrical conductivity of leaves (REC) was calculated as REC (%) = R1/R2 × 100 [42].

### 3.4. Quantitative Gene Expression Level by Real-Time PCR Analysis

To verify the microarray results and compare genes expression patterns between parental genotypes and NILs, total RNAs of the collected samples were extracted using TRIzol reagent (Invitrogen). First-strand cDNA was synthesized using Superscript III reverse transcriptase (Invitrogen), according to the manufacturer’s instruction. Real-time PCR was conducted on an ABI Prism 7500 real-time PCR system (Applied Biosystems). For real-time PCR analysis, the gene-specific primers were designed by Primer Express Version 2.0 (Applied Biosystems) (Table S1). The expression level of rice Profilin-2 gene (*LOC_Os06g05880*), which is expressed stably in most tissues or organs [23] and under different abiotic stress conditions in our study, was used as the internal control. Real-time PCR amplifications were performed in an optical 96-well plate. Each reaction was done in a volume of 25 μL containing 12.5 μL of 2× SYBR green master reagent (Applied Biosystems), 5.0 μL diluted transcription product and 0.2 μL of each gene-specific primer. The following thermal cycle was used: 95 °C for 3 min and then 45 cycles of 95 °C for 30 s, 60 °C for 30 s and 72 °C for 1 min. Dissociation curve analysis was performed using the following thermal profile: 95 °C for 15 s, 60 °C for 20 s and 95 °C for 15 min. The relative expression levels were determined, as described previously [43].

### 3.5. Accession Numbers

The microarray data of two rice varieties have been deposited in the NCBI/GEO database, and the GEO accession number is GSE25176.

## 4. Conclusions

Upland and paddy rice have distinctive features in response to drought stress. Our findings based on morphological and physiological comparison of two genotypes representing upland and paddy rice suggest that upland rice responds to the early drought stress more efficiently than paddy rice, mainly through drought-avoidance mechanisms, such as active leaf rolling (avoiding rapid water loss) and root growth (absorbing more water). Comparative expression profiling analysis of the two genotypes has provided some explanations of the differential drought responses at the genomic expression level. Drought resistance is such a complex trait that comparing the expression profiles between only two parent lines with distinct genetic backgrounds provides limited information on the drought-response mechanisms. However, as shown in this study, comparison of the expression patterns of drought-responsive genes between the parents and the NILs of targeted QTL can provide valuable information in dissecting the genetic bases of the drought-resistance traits, as well as in narrowing the candidate genes for the traits.

## Supplementary Information



## Figures and Tables

**Table 1:** Phenotypic difference of ZS97 and IRAT109 under natural and drought stress.

Trait	Normal growth		Drought stress		Relative value b	
		
	ZS97	IRAT109	ZS97	IRAT109	ZS97	IRAT109
PH	87.34 ± 6.96	103.95 ± 7.58 ^**^^a^	80.81 ± 6.63	95.33 ± 6.63 ^**^	0.926	0.917
GY	41.72 ± 5.41 ^*^	38.45 ± 7.11	19.83 ± 7.4	25.55 ± 10.14 ^**^	0.475	0.662
TPP	31.57 ± 2.27 ^**^	24.85 ± 2.13	23.84 ± 8.12 ^**^	13.13 ± 5.94	0.753	0.526
SPP	93.34 ± 17.81	114.18 ± 24.94 ^**^	75.44 ± 12.57 ^**^	72.14 ± 16.51	0.808	0.631
SF	0.91 ± 0.06 ^**^	0.67 ± 0.08	0.47 ± 0.10	0.46 ± 0.20	0.516	0.687
KGW	23.48 ± 2.02	32.28 ± 2.14 ^**^	19.69 ± 2.27	31.26 ± 3.84 ^**^	0.838	0.969
RV	36.62 ± 9.43	60.51 ± 10.27 ^**^	20.82 ± 8.43	31.56 ± 9.15 ^**^	0.568	0.522
RVT	1.16 ± 0.37	2.43 ± 0.57 ^**^	0.86 ± 0.27	2.38 ± 0.50 ^**^	0.741	0.979

a The difference between ZS97 and IRAT109: ^*^ and ^**^significant at *p* = 0.05 and *p* = 0.01 level.

b Relative value indicated the ratio trait value under normal growth/drought stress.

GY: grain yield; PH: plant height; KGW: 1000-grain weight; RV: total root volumes; RVT: root volumes per tiller; SF: spikelet fertility; SPP: number of spikelets per panicle; TPP: tillers per plant.

**Table 2:** Comparison of function categories of drought-responsive genes.

GO.ID	Term	IRAT109				ZS97		
	
		Level a	Significant b	Expected c	Classic d	Significant b	Expected c	Classic d
Up								

GO:0006950	response to stress	3	233	180.79	9.60 × 10^−6^	250	180.61	8.50 × 10^−9^
GO:0009605	response to external stimulus	3	69	43.5	8.10 ×10^−5^	80	43.46	5.90×10^−8^
GO:0005975	carbohydrate metabolic process	4	111	77.88	7.00 ×10^−5^	131	77.8	1.00 × 10^−9^
GO:0009991	response to extracellular stimulus	4	16	7.84	0.00475	21	7.83	2.60 × 10^−5^
GO:0000271	polysaccharide biosynthetic process	6	10	4.3	0.00949	12	4.29	0.00093
GO:0042221	response to chemical stimulus	3	-	-	-	32	19.36	0.00338
GO:0006725	cellular aromatic compound metabolic process	4	-	-	-	24	12.88	0.00225
GO:0017144	drug metabolic process	4	-	-	-	10	3.54	0.00224
GO:0016051	carbohydrate biosynthetic process	5	-	-	-	19	9.49	0.00275
GO:0016999	antibiotic metabolic process	5	-	-	-	10	3.54	0.00224
GO:0044270	nitrogen compound catabolic process	5	-	-	-	7	2.18	0.00483
GO:0017000	antibiotic biosynthetic process	6	-	-	-	8	3.01	0.00889
GO:0034637	cellular carbohydrate biosynthetic process	6	-	-	-	13	5.65	0.00369
GO:0009250	glucan biosynthetic process	8	-	-	-	10	3.84	0.0042
GO:0016787	hydrolase activity	3	-	-	-	290	246.07	0.00093
GO:0016491	oxidoreductase activity	3	-	-	-	53	37.37	0.0065
GO:0006139	nucleobase, nucleoside, nucleotide and nucleic acid metabolic process	4	286	250.98	0.00506	-	-	-
GO:0022402	cell cycle process	4	6	1.36	0.00153	-	-	-
GO:0044262	cellular carbohydrate metabolic process	5	24	13.95	0.00629	-	-	-
GO:0016311	dephosphorylation	6	13	5.81	0.0047	-	-	-
GO:0044042	glucan metabolic process	6	13	6.11	0.00727	-	-	-
GO:0006073	cellular glucan metabolic process	7	13	6.11	0.00727	-	-	-
GO:0006350	transcription	7	187	145.2	9.70 × 10^−5^	-	-	-
GO:0006470	protein amino acid dephosphorylation	9	13	5.81	0.0047	-	-	-
GO:0045735	nutrient reservoir activity	2	11	4.78	0.00753	-	-	-
GO:0003676	nucleic acid binding	3	325	286.7	0.00509	-	-	-

Down								
GO:0050896	response to stimulus	2	608	465.77	2.90 × 10^−17^	583	447.76	2.80 × 10^−16^
GO:0065007	biological regulation	2	441	378	4.30 × 10^−5^	416	363.39	0.00042
GO:0022414	reproductive process	3	82	52.03	1.90 ×10^−5^	71	50.02	0.0015
GO:0048856	anatomical structure development	3	73	46.77	7.40 × 10^−5^	66	44.96	0.00092
GO:0006793	phosphorus metabolic process	4	252	158.57	3.70 × 10^−5^	231	152.44	1.10 ×10^−11^
GO:0009908	flower development	6	66	40.61	4.80 × 10^−5^	60	39.04	0.00049
GO:0016310	phosphorylation	6	242	147.35	2.90 × 10^−16^	221	141.65	1.90 × 10^−12^
GO:0043687	post-translational protein modification	8	278	194.01	3.50 × 10^−11^	255	186.51	2.70 ×10^−8^
GO:0006468	protein amino acid phosphorylation	9	233	136.72	1.40 × 10^−17^	215	131.44	3.00 × 10^−14^
GO:0009653	anatomical structure morphogenesis	4	50	26.71	9.20 × 10^−6^	42	25.68	0.00096
GO:0030154	cell differentiation	4	83	49.94	2.10 × 10^−6^	76	48.01	3.40 ×10^−5^
GO:0048608	reproductive structure development	5	71	43.49	2.10 × 10^−5^	64	41.81	0.00036
GO:0006464	protein modification process	7	340	266.3	1.50 × 10^−7^	307	256	0.00014
GO:0006725	cellular aromatic compound metabolic process	4	-	-	-	27	16.32	0.00619
GO:0006979	response to oxidative stress	4	-	-	-	27	15.65	0.00347
GO:0009991	response to extracellular stimulus	4	-	-	-	18	9.93	0.00921
GO:0046164	alcohol catabolic process	4	-	-	-	21	12.22	0.00959
GO:0009225	nucleotide-sugar metabolic process	5	-	-	-	18	8.11	0.00094
GO:0019318	hexose metabolic process	6	-	-	-	24	13.75	0.00482
GO:0046365	monosaccharide catabolic process	6	-	-	-	21	11.74	0.00608
GO:0007155	cell adhesion	3	9	3.67	0.00876	-	-	-
GO:0006810	transport	4	222	188.16	0.00338	-	-	-
GO:0006811	ion transport	5	63	40.11	0.00019	-	-	-
GO:0015837	amine transport	5	18	8.54	0.00171	-	-	-
GO:0015849	organic acid transport	5	18	8.74	0.00224	-	-	-
GO:0055085	transmembrane transport	5	31	19.16	0.00487	-	-	-
GO:0006812	cation transport	6	49	34.65	0.00803	-	-	-
GO:0006820	anion transport	6	11	4.57	0.00452	-	-	-
GO:0006855	multidrug transport	6	10	4.37	0.00966	-	-	-
GO:0009309	amine biosynthetic process	6	24	14.3	0.00781	-	-	-
GO:0006865	amino acid transport	7	18	8.54	0.00171	-	-	-
GO:0015698	inorganic anion transport	7	10	3.08	0.00057	-	-	-
GO:0008652	cellular amino acid biosynthetic process	8	22	12.81	0.00809	-	-	-
GO:0009064	glutamine family amino acid metabolic process	8	10	3.97	0.00475	-	-	-
GO:0009069	serine family amino acid metabolic process	8	11	4.96	0.00876	-	-	-
GO:0004553	hydrolase activity, hydrolyzing *O*-glycosyl compounds	5	47	32.8	0.00784	-	-	-

a Level of Term in gene ontology.

b The number of loci of the GO term observed.

c The number of loci expected.

d *p*-value of Fisher’s exact test.

**Table 3:** Comparison of expression patterns of 36 drought-responsive genes.

TIGR Locus	Annotation (TIGR version 6.1)	Normal a				Drought b					
	
		ZS97	IRAT109	NIL-ZS	NIL-IR	ZS97	IRAT109	NIL-ZS	NIL-IR	C1 c	C2 d
LOC_Os02g32250	retrotransposon protein	−	+	−	+	↑	↑	↑	↑	4	a
LOC_Os02g40240	receptor kinase	+	−			↓	↓		↑	1	d
LOC_Os02g41710	cyclic nucleotide-gated ion channel	+	−	+	−	↑	↑	↓	↑	2	d
LOC_Os02g44780	polyprenyl synthetase					↑	↑	↑	↑	4	a
LOC_Os02g48360	pyrophosphate-fructose 6-phosphate 1-phosphotransferase subunit alpha	−	+	−	+	↑	↑	↑	↑	4	a
LOC_Os02g48710	expressed protein			+	−	↑		↑	↑	3	b
LOC_Os02g51840	expressed protein			+	−				↑	3	d
LOC_Os02g53200	glucan endo-1,3-beta-glucosidase precursor	+	−	+	−	↓	↑	↓	↑	4	c
LOC_Os02g54254	saccharopine dehydrogenase	+	−			↑	↑	↑	↑	4	a
LOC_Os03g03034	flavonol synthase/flavanone 3-hydroxylase			−	+	↑	↑		↓	1	d
LOC_Os03g03050	expressed protein	+	−			↑	↑	↑	↑	4	a
LOC_Os03g03790	AMP-binding domain containing protein			−	+	↓	↓	↓	↓	4	a
LOC_Os03g36550	transposon protein	+	−			↑	↑		↑	2	d
LOC_Os03g37960	acyl CoA binding protein					↑			↑	1	c
LOC_Os03g38800	AAA family ATPase	−	+				↓	↓	↓	2	b
LOC_Os04g07890	AGAP002737-PA	+	−			↑	↑		↑	2	d
LOC_Os04g08280	retrotransposon protein,Ty3-gypsy subclass	+	−	−	+	↑	↑	↑	↓	3	d
LOC_Os04g08800	expressed protein	−	+			↑	↑	↑	↑	4	a
LOC_Os04g52450	aminotransferase	+	−	+	−		↑		↑	4	c
LOC_Os04g52640	SHR5-receptor-like kinase	−	+	−	+	↑	↑		↓	1	d
LOC_Os04g52670	OsSAUR21 - Auxin-responsive SAUR gene family member	+	−	+	−		↑		↑	4	c
LOC_Os05g37450	starch binding domain containing protein	−	+			↑		↑	↑	3	b
LOC_Os05g37830	expressed protein	+	−				↑	↑	↑	2	b
LOC_Os05g38360	DHHC zinc finger domain containing protein	+	−				↑			3	b
LOC_Os07g10970	leucine zipper protein-like	−	+					↓	↑	1	c
LOC_Os07g15770	CCT motif family protein	−	+	−	+	↓	↑	↑		1	c
LOC_Os09g13440	expressed protein	+	−	+	−	↑↓		↓	↑	1	c
LOC_Os09g14450	pollen signaling protein with adenylyl cyclase activity	−	+				↓		↑	3	c
LOC_Os11g29790	receptor kinase	−	+			↑	↓	↓	↓	2	b
LOC_Os11g35274	protein kinase domain containing protein	+	−	+	−		↑	↑	↑	2	b
LOC_Os12g03740	OsFBX438 - F-box domain containing protein					↑	↑	↑	↑	4	a
LOC_Os12g07280	ZOS12-02 - C2H2 zinc finger protein	−	+	−	+	↓		↓↑	↓	1	c
LOC_Os12g08260	dehydrogenase E1 component domain containing protein	+	−			↑	↑	↑	↑	4	a
LOC_Os12g39360	aspartic proteinase nepenthesin precursor	+	−			↓	↓	↓	↓	4	a
LOC_Os12g39520	OsFBDUF66 - F-box and DUF domain containing protein	+	−			↑	↑	↑	↑	4	a
LOC_Os12g44100	peptide transporter PTR2	+	−			↓	↑	↓	↓	3	b

a Comparison of expression patterns under normal condition. − and + indicate significant different expression level between ZS97 and IRAT109 or between NIL-ZS and NIL-IR, −: lower expression level and +: higher expression level.

b Comparison of expression patterns under drought condition. ↑: upregulated by drought stress; ↓: downregulated by drought stress; ↑↓: expression level was first increased and then decreased; ↓↑: expression level was first decreased and then increased.

c C1: Four groups (1–4) classified based on the difference in responsive trend between the parent line and the corresponding NIL.

d C2: Four groups (a–d) classified based on the difference in responsive trend to drought between the two parents and/or between the paired NILs.
