# Supplementary material for: Insight into Differential Responses of Upland and Paddy Rice to Drought Stress by Comparative Expression Profiling Analysis
Source: Int J Mol Sci. 2013 Mar 4;14(3):5214–38. doi: 10.3390/ijms14035214 (PMC3634487; doi:10.3390/ijms14035214)
Supplement: Supplementary file 1 [file ijms-14-05214-s001.doc]

Supplementary Information

**Figure S1.** Reliability of microarray data. (**A**) The correlation coefficients of biological replicates and time points. IR and ZS: IRAT109 and ZS97. IR-0, IR-1, IR-2, and IR-3: four time points in IRAT109. IR-0-1 and IR-0-2: two biological replicates; (**B**) Verification of microarray results with real-time RT-PCR. Transcriptions ratios determined from the microarray hybridization were ln-transformed and plotted against those obtained from real-time RT-PCR analysis. R: Pearson correlation coefficient.

**
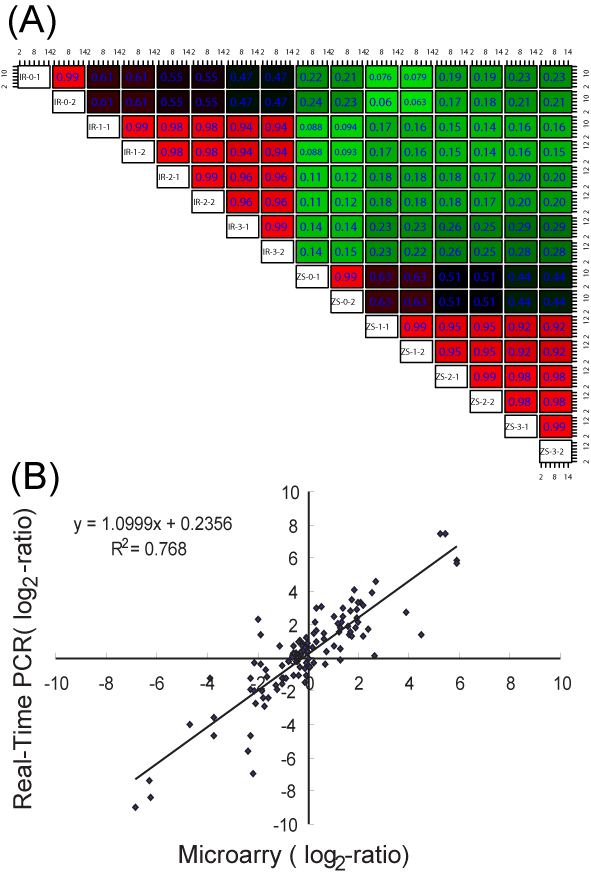
**

**Table S1.** Primers for real-time PCR analyses of drought-responsive genes and internal controls.

| **NILs** | **Loucs ID** | **Primers for real-time PCR** | |
| --- | --- | --- | --- |
|  |  | **Forward primers** | **Reverse primers** |
| N4 | LOC_Os02g32250 | AGGGTTAGAGCACGACACCAA | GCGGCGGCTAAAGGTATAAGA |
| N6 | LOC_Os02g40240 | AAAATGCTGTCCACCAGTGCTT | CACTTGGTAATTTGCGCGTACA |
|  | LOC_Os02g41710 | CAATATGCAAGCGACGATGGT | TTCATCCATCGCTCCATGTC |
|  | LOC_Os02g44780 | GCTGCAGGATGCAGCTACAA | CAAATATCCGGTAGCATCAAGGT |
| N8 | LOC_Os02g48360 | CCAGGAATACTTGGAGAAGGTGAA | AGCGCTGCCTTGAGAACATC |
|  | LOC_Os02g48710 | CGGGTGGGATCATGCACTA | CGGAGATCATCAACGAACCAAT |
| N9 | LOC_Os02g51840 | GCAGATCGGTCTCGCATAGC | CCAACCGCCCAAACACA |
|  | LOC_Os02g53200 | TTTTCCAGTTTTCCTCCCTTTCT | CAAACATGCAGCAGGAGTCAA |
|  | LOC_Os02g54254 | TGGAAGGAAATGGTGTGATCTG | AGAGCCCACCTCTTTGGAAAA |
| N11 | LOC_Os03g03034 | GGATGTCCGTCGCATCGT | CCATCGGTGATGAGCTTCTGA |
|  | LOC_Os03g03050 | AGAGATAGGCACCCCCCTGAT | TGCTAGGTCCTCCCTTCTTCCT |
|  | LOC_Os03g03790 | GTGTCAAGGCTTTAGGGCAATC | TCCTACGTGCAAAAGGTGATATCA |
| N12 | LOC_Os03g36550 | GGCAATTATGACTTGGGATTTTCTA | CTTCCTCCACAACTAAAAGCTGATT |
|  | LOC_Os03g37960 | TCGGACACAAATGAAAAAACAAA | CACATGTGCTAATGACGGCTTAA |
|  | LOC_Os03g38800 | CGTGGAGTTCCTCCGTGAGA | AAGCTGCATTGCCTTTTTCTG |
| N15 | LOC_Os04g07890 | AGGACAGGGTGTGGGAGTTG | CTGCCGCTTTTGTCTCCAA |
|  | LOC_Os04g08280 | GACGCCAGCAGCACGTT | AATTAAGATCGAGGGCAGAAGGT |
|  | LOC_Os04g08800 | GCCCAGTCTCTTGGATGTTTTAA | GGTTGCCAGCAAACATTTTTG |
| N19 | LOC_Os04g52450 | GACCGAGTTGACAACTCCTCATC | TCATCGCTGCCAGCATTATC |
|  | LOC_Os04g52640 | TCAAGGGCAAATGCGTTTTT | CGGTTCAAGTTTATCCTCCCTTT |
|  | LOC_Os04g52670 | CCTCGCGCTCTCCTACTTCA | GGATCAACGCTACGCTACTACATG |
| N23 | LOC_Os05g37450 | GTGCTTTTGAACATGAGTTGCTTT | GCAGTACCTTATGACTCGTTCATGA |
|  | LOC_Os05g37830 | CTACAACGGGCTCAATTGCAT | CATTGGGTCTTTCCACTTTGATAA |
|  | LOC_Os05g38360 | CTGGCTTTGTCCAGTATCGACTAA | CGTGTTAATCACATCGATGGTGTA |
| N24 | LOC_Os07g10970 | ACATGAAGTCAGCGCTGCAA | GTGCTCAAGGTATCTCGCTATCTG |
|  | LOC_Os07g15770 | GAAGAGGTGCTACGAGAAGCAAA | GGCGAAGCGACCTCTCACT |
| N29 | LOC_Os09g13440 | ATGCTACATCAAGAGTGGGAGCTA | AGGTGGACGGTCCTCTTGGT |
|  | LOC_Os09g14450 | ACCGAGGTGCTACTCGACAGA | ACATGTTCAGCAGCAGCTTGA |
| N36 | LOC_Os11g29790 | GAGCCTGGATAGGTGGTTACATCT | GGCAATGTTCAATCTCTGGATCA |
|  | LOC_Os11g35274 | AGTTCCGTAGCAGAGTGGCAAT | CTCCAAACAAATCCCCAGAAGT |
| N37 | LOC_Os12g03740 | CCAAGGAGATGGAATGGTTGTT | ACCCTCGAGGATAAGTCCTTTCTT |
|  | LOC_Os12g07280 | GATTTCGGATTCGGGCAGTT | GATCACCCGCGAATTGCT |
|  | LOC_Os12g08260 | GTCGGCCATCACTCGACAT | CTGTTCGCCAGTGCTCGAT |
| N38 | LOC_Os12g39360 | GCAACTACCAGCAGCAGAACAT | GGCAGGCTGGAAGGAGAAGT |
|  | LOC_Os12g39520 | CTACGACAATCCGACGAAAGAGT | CATGTTGTACACGCCAGAGTCA |
|  | LOC_Os12g44100 | GCGTCGACCTCTTCTACCTTGT | CGATGTTCTTGGACCTGTACCA |
|  | LOC_Os06g05880 | TGGCCTGTCGTTGATTCTTG | CCTCTCCACAATCATGTTGCA |

**Table S2.** Transcript level stability check of 24 genes for selecting internal control genes for quantitative expression analysis under drought stress.

| **Probe set** | **Locus ID** | **mean** | **CV** | **Annotation** |
| --- | --- | --- | --- | --- |
| Os.28425.1.S1_x_at | LOC_Os12g43600 | 13403.9 | 12.69% | Glycine-rich RNA-binding protein GRP1A |
| Os.12237.1.S1_a_at | LOC_Os06g47230 | 8310.13 | 9.59% | Expressed protein |
| Os.10152.1.S1_at | LOC_Os06g05880 | 6763.71 | 13.06% | Profilin-2 |
| Os.10931.1.S1_at | LOC_Os05g41060 | 7601.79 | 11.22% | ADP-ribosylation factor |
| Os.12602.1.S1_at | LOC_Os01g05490 | 5783.48 | 14.65% | Triosephosphate isomerase |
| Os.46231.1.S1_a_at | LOC_Os03g46770 | 11012.5 | 22.90% | RNA recognition motif containing protein |
| Os.4157.1.S1_at | LOC_Os02g02890 | 10788.7 | 16.85% | Peptidyl-prolyl cis-trans isomerase |
| Os.7897.1.S1_at | LOC_Os08g27850 | 7910.59 | 15.57% | Endothelial differentiation-related factor 1 |
| Os.10139.1.S1_s_at | LOC_Os06g46770 | 7454.61 | 33.12% | polyubiquitin containing 7 ubiquitin monomers |
| Os.7945.1.S1_at | LOC_Os07g34589 | 13465.1 | 14.10% | protein translation factor SUI1 |
| Os.12168.2.S1_s_at | LOC_Os08g03290 | 7301.97 | 20.78% | GAPDH |
| Os.22660.3.S1_x_at | LOC_Os02g06640 | 9282.62 | 21.63% | polyubiquitin containing 7 ubiquitin monomers |
| Os.4705.1.S1_at | LOC_Os02g32030 | 4120.06 | 22.37% | Elongation factor 2 |
| Os.318.1.S1_at | LOC_Os03g55150 | 4589.46 | 25.84% | Eukaryotic translation initiation factor 5A-2 |
| Os.11479.1.S1_at | LOC_Os01g60410 | 3811.1 | 46.36% | Ubiquitin-conjugating enzyme E2 |
| Os.8152.1.S1_at | LOC_Os05g49890 | 5399.44 | 25.83% | GTP-binding nuclear protein Ran/TC4 |
| Os.12749.1.S1_s_at | LOC_Os03g60590 | 4223.26 | 21.15% | Actin-depolymerizing factor 3 |
| Os.12178.1.S1_at | LOC_Os02g52290 | 5487.62 | 30.86% | Peptidylprolyl isomerase FKBP12 |
| Os.4746.1.S1_a_at | LOC_Os02g48660 | 5138.55 | 19.33% | 60S ribosomal protein L31 |
| Os.12625.2.S1_x_at | LOC_Os03g08020 | 5231.01 | 25.92% | Elongation factor 1-alpha |
| Os.12772.1.S1_at | LOC_Os03g13170 | 4038.76 | 29.63% | ubiquitin fusion protein |
| Os.3420.1.S1_s_at | LOC_Os03g50890 | 1512.52 | 57.64% | Actin-1 |
| Os.22781.1.S1_at | LOC_Os02g38920 | 1650.82 | 43.77% | GAPDH |
| Os.7916.1.S1_at | LOC_Os01g59150 | 961.547 | 74.72% | Tubulin beta-6 chain |

**Table S3.** Number of drought-responsive genes in 17 intervals of drought-resistance QTL.

| **NILs** | **Genomic background** | **Induced** | | **Repressed** | | **NO. of ORFs** |
| --- | --- | --- | --- | --- | --- | --- |
|  |  | **IRAT109** | **ZS97** | **IRAT109** | **ZS97** |  |
| N01 | ZS97 | 9 | 3 | 7 | 9 | 577 |
| N04 | IRAT109 | 6 | 0 | 0 | 3 | 411 |
| N06 | ZS97 | 3 | 2 | 7 | 12 | 572 |
| N08 | ZS97 | 2 | 2 | 4 | 6 | 531 |
| N09 | ZS97 | 4 | 4 | 4 | 3 | 452 |
| N11 | IRAT109 | 2 | 0 | 4 | 1 | 139 |
| N12 | IRAT109 | 2 | 1 | 4 | 1 | 418 |
| N15 | IRAT109 | 3 | 2 | 2 | 3 | 565 |
| N19 | ZS97 | 5 | 3 | 4 | 1 | 293 |
| N20 | ZS97 | 0 | 1 | 0 | 2 | 315 |
| N23 | ZS97 | 3 | 5 | 2 | 8 | 653 |
| N24 | ZS97 | 2 | 4 | 4 | 3 | 521 |

**Table S3.** *Cont.*

| N29 | ZS97 | 2 | 0 | 2 | 0 | 399 |
| --- | --- | --- | --- | --- | --- | --- |
| N30 | ZS97 | 1 | 3 | 6 | 3 | 561 |
| N36 | IRAT109 | 3 | 3 | 7 | 4 | 519 |
| N37 | IRAT109 | 4 | 4 | 2 | 0 | 647 |
| N38 | IRAT109 | 3 | 1 | 0 | 3 | 470 |
| total |  | 54 | 38 | 59 | 62 | 8043 |

**Table S4.** List of 213 drought-responsive genes in 17 intervals of drought-resistance QTL.

| **Probe sets** | **Annotation** |
| --- | --- |
| Os.12253.1.S1_at | anthocyanin 3-O-beta-glucosyltransferase, putative, expressed |
| Os.11247.1.S1_at | cinnamoyl-CoA reductase-related, putative, expressed |
| Os.12629.1.S1_at | expressed protein |
| Os.4767.2.S1_x_at | ketol-acid reductoisomerase, chloroplast precursor, putative, expressed |
| Os.41454.1.S1_s_at | DUF1645 domain containing protein, putative, expressed |
| Os.10006.1.S1_at | OsMan01 - Endo-Beta-Mannanase, expressed |
| Os.33575.1.S1_at | serine/threonine-protein kinase, putative, expressed |
| Os.14999.3.S1_x_at | CGMC_MAPKCMGC_2.6 - CGMC includes CDA, MAPK, GSK3, and CLKC kinases, expressed |
| OsAffx.9318.1.S1_at | expressed protein |
| Os.52073.1.S1_at | HMG1/2, putative, expressed |
| Os.10135.1.S1_at | splicing factor-related, putative, expressed |
| Os.35583.1.S1_at | expressed protein |
| Os.10715.1.S1_a_at | expressed protein |
| Os.9943.5.S1_x_at | OsWAK10d - OsWAK receptor-like cytoplasmic kinase OsWAK-RLCK, expressed |
| Os.11986.2.S1_x_at | Protein kinase domain containing protein, expressed |
| Os.8019.1.S1_at | B3 DNA binding domain containing protein, expressed |
| Os.47360.1.S1_x_at | protein kinase, putative, expressed |
| Os.41637.1.S1_at | MDR-like ABC transporter, putative, expressed |
| Os.39411.1.A1_at | ABC transporter, ATP-binding protein, putative, expressed |
| Os.45892.1.S1_at | MYB family transcription factor, putative, expressed |
| Os.47750.1.A1_at | Cupin domain containing protein, expressed |
| OsAffx.12287.1.S1_at | conserved hypothetical protein |
| Os.36104.1.S1_at | glycerophosphoryl diester phosphodiesterase family protein, putative, expressed |
| Os.14835.2.S1_at | hydroxyproline-rich glycoprotein family protein, putative, expressed |
| Os.46107.1.S1_s_at | retrotransposon protein, putative, unclassified, expressed |
| Os.27096.1.S1_at | SAM domain family protein, expressed |
| Os.49660.1.S1_at | expressed protein |

***Table S4.*** *Cont.*

| Os.12094.1.S1_a_at | abscisic stress-ripening, putative, expressed |
| --- | --- |
| OsAffx.2912.1.S1_at | RNA recognition motif containing protein, putative, expressed |
| Os.57134.1.S1_at | expressed protein |
| OsAffx.2924.1.S1_at | receptor-like protein kinase 5 precursor, putative, expressed |
| OsAffx.12425.1.S1_at | receptor kinase, putative |
| Os.52563.2.S1_x_at | receptor-like protein kinase precursor, putative, expressed |
| Os.40418.1.S1_at | receptor kinase, putative, expressed |
| Os.53830.1.S1_at | alcohol oxidase, putative, expressed |
| Os.46154.1.S1_s_at | aquaporin protein, putative, expressed |
| Os.52644.1.S1_at | phytosulfokine receptor precursor, putative, expressed |
| Os.8499.1.S1_a_at | OsSCP8 - Putative Serine Carboxypeptidase homologue, expressed |
| Os.53236.1.S1_at | B-box zinc finger family protein, putative, expressed |
| Os.5542.1.S1_at | cytochrome b5-like Heme/Steroid binding domain containing protein, expressed |
| Os.1327.1.S1_at | retrotransposon protein, putative, unclassified, expressed |
| Os.53660.1.S1_at | ethylene-responsive transcription factor, putative, expressed |
| Os.1352.1.S1_at | expressed protein |
| Os.14380.1.S1_at | MSP domain containing protein, expressed |
| Os.21578.1.S1_at | scarecrow, putative, expressed |
| Os.14092.1.S1_at | expressed protein |
| Os.18321.2.S1_at | polyprenyl synthetase, putative, expressed |
| Os.14076.1.S1_s_at | rho-GTPase-activating protein-related, putative, expressed |
| OsAffx.24733.1.S1_s_at | RALFL8 - Rapid ALkalinization Factor RALF family protein precursor, expressed |
| Os.53431.1.S1_at | expressed protein |
| Os.49582.1.S1_at | expressed protein |
| Os.4377.1.S1_at | AMP-binding domain containing protein, expressed |
| Os.11658.1.S1_at | nuclear matrix constituent protein 1-like, putative, expressed |
| Os.12866.1.S1_at | pyrophosphate--fructose 6-phosphate 1-phosphotransferase subunit alpha, putative, expressed |
| Os.5592.1.S1_at | expressed protein |
| Os.7909.1.S1_at | glutamine synthetase, catalytic domain containing protein, expressed |
| OsAffx.12575.1.S1_at | RNA-dependent RNA polymerase, putative, expressed |
| Os.17509.1.S1_at | endoglucanase, putative, expressed |
| Os.55360.1.S1_at | expressed protein |
| Os.6170.1.S1_at | CSLA6 - cellulose synthase-like family A; mannan synthase, expressed |
| Os.52106.2.S1_at | mTERF family protein, expressed |
| Os.47984.1.A1_at | expressed protein |

***Table S4.*** *Cont.*

| Os.5659.1.S1_at | OsFBX61 - F-box domain containing protein, expressed |
| --- | --- |
| Os.25104.1.S1_x_at | expressed protein |
| Os.47727.1.S1_s_at | uncharacterized Cys-rich domain containing protein, putative, expressed |
| Os.51673.1.S1_at | cold-induced protein, putative, expressed |
| Os.11714.1.S1_at | glucan endo-1,3-beta-glucosidase precursor, putative, expressed |
| Os.10695.1.S1_at | adenylate kinase, putative, expressed |
| OsAffx.24862.1.S1_at | OsFBX62 - F-box domain containing protein |
| Os.52005.1.S1_at | saccharopine dehydrogenase, putative, expressed |
| OsAffx.24875.1.S1_at | expressed protein |
| Os.48072.1.S1_at | MCM5 - Putative minichromosome maintenance MCM complex subunit 5, expressed |
| Os.49445.1.S1_at | F-box/LRR-repeat protein 14, putative, expressed |
| Os.20333.1.S1_at | expressed protein |
| Os.25112.1.S1_at | methyltransferase, putative, expressed |
| Os.33603.1.S1_at | heavy metal-associated domain containing protein, expressed |
| Os.49527.1.S1_at | AMP-binding domain containing protein, expressed |
| Os.21349.1.S1_at | expansin precursor, putative, expressed |
| Os.52832.1.S1_at | CorA-like magnesium transporter protein, putative, expressed |
| Os.48829.1.A1_at | cytochrome P450 86A1, putative, expressed |
| Os.22677.2.S1_at | magnesium-chelatase subunit chlI, chloroplast precursor, putative, expressed |
| OsAffx.25404.1.S1_at | hypothetical protein |
| OsAffx.3427.1.S1_at | acyl CoA binding protein, putative |
| Os.54109.1.S1_at | AAA family ATPase, putative, expressed |
| Os.33081.1.S1_at | expressed protein |
| Os.54563.1.S1_at | OsSub30 - Putative Subtilisin homologue, expressed |
| OsAffx.13282.1.S1_s_at | DUF260 domain containing protein, putative, expressed |
| Os.27402.1.S1_at | disease resistance protein, putative, expressed |
| Os.54228.1.S1_at | OsWAK29 - OsWAK receptor-like protein kinase, expressed |
| Os.9739.1.S1_at | expressed protein |
| Os.6075.1.S2_at | expressed protein |
| OsAffx.13717.1.S1_s_at | AGAP002737-PA, putative, expressed |
| Os.55383.1.S1_at | retrotransposon protein, putative, Ty3-gypsy subclass, expressed |
| Os.54321.1.S1_at | expressed protein |
| Os.6217.1.S2_at | expressed protein |
| Os.34698.1.S1_x_at | aldose 1-epimerase, putative, expressed |
| Os.50175.2.S1_at | OsHKT1;1 - Na+ transporter, expressed |
| Os.10635.1.S1_s_at | GHMP kinases ATP-binding protein, putative, expressed |

***Table S4.*** *Cont.*

| Os.12293.1.S1_a_at | expressed protein |
| --- | --- |
| Os.27084.2.S1_a_at | SHR5-receptor-like kinase, putative, expressed |
| OsAffx.14380.1.S1_s_at | OsSAUR21 - Auxin-responsive SAUR gene family member, expressed |
| Os.50342.1.S1_at | leucine-rich repeat receptor protein kinase EXS precursor, putative, expressed |
| Os.22807.1.S1_s_at | NBS-LRR disease resistance protein, putative, expressed |
| Os.23474.1.A1_at | RNA recognition motif containing protein, putative, expressed |
| OsAffx.14407.1.S1_at | ARK3, putative |
| Os.9913.1.S1_at | wound induced protein, putative, expressed |
| Os.8482.1.S1_s_at | LTPL125 - Protease inhibitor/seed storage/LTP family protein precursor, putative, expressed |
| OsAffx.14425.1.S1_at | coatomer subunit epsilon, putative, expressed |
| Os.7692.1.S1_at | tetratricopeptide repeat domain containing protein, putative, expressed |
| Os.54080.1.S1_at | starch binding domain containing protein, putative, expressed |
| Os.18881.1.S1_at | periplasmic beta-glucosidase precursor, putative, expressed |
| Os.14077.1.S1_at | expressed protein |
| OsAffx.15014.1.S1_s_at | plant-specific domain TIGR01589 family protein, expressed |
| Os.23209.1.S1_at | PMR5, putative, expressed |
| Os.18913.2.S1_at | NFD4, putative, expressed |
| Os.6671.2.S1_x_at | spotted leaf 11, putative, expressed |
| Os.4820.1.S1_at | expressed protein |
| Os.6676.1.S1_at | NADH-cytochrome b5 reductase, putative, expressed |
| Os.15191.1.S1_s_at | SNF1-related protein kinase regulatory subunit beta-1, putative, expressed |
| Os.52922.1.S1_at | SH2 motif, putative, expressed |
| OsAffx.15062.1.S1_at | ribosomal protein L11 methyltransferase-related, putative, expressed |
| Os.28129.1.S1_at | UDP-glucoronosyl and UDP-glucosyl transferase domain containing protein, expressed |
| OsAffx.27284.1.S1_s_at | cyclin-B1-1, putative, expressed |
| Os.49209.1.S1_at | expressed protein |
| Os.51231.1.S1_at | cytochrome P450, putative, expressed |
| OsAffx.27304.1.S1_x_at | trehalose-6-phosphate synthase, putative, expressed |
| Os.52491.1.A1_at | expressed protein |
| OsAffx.5290.1.S1_at | leucine zipper protein-like, putative, expressed |
| Os.30746.1.S1_at | cytochrome P450, putative, expressed |

***Table S4.*** *Cont.*

| OsAffx.28409.1.S1_at | EF hand family protein, putative |
| --- | --- |
| OsAffx.10193.1.S1_at | hypothetical protein |
| Os.28865.1.S1_at | expressed protein |
| Os.26753.1.S1_at | expressed protein |
| Os.14631.1.S1_at | UDP-glucoronosyl and UDP-glucosyl transferase domain containing protein, expressed |
| OsAffx.16247.1.S1_at | cytokinin-N-glucosyltransferase, putative, expressed |
| Os.35797.1.S1_at | OsFBX230 - F-box domain containing protein, expressed |
| OsAffx.28463.1.S1_at | pentatricopeptide, putative, expressed |
| Os.5744.1.S1_at | phospholipase D, putative, expressed |
| Os.50228.1.S1_at | legume lectins beta domain containing protein, expressed |
| Os.12579.1.S1_at | acetyl-CoA acetyltransferase, cytosolic, putative, expressed |
| OsAffx.29770.1.S1_at | cinnamoyl CoA reductase, putative, expressed |
| Os.50376.2.S1_at | nuclear transport factor, putative, expressed |
| OsAffx.17614.1.S1_at | disease resistance protein RPM1, putative, expressed |
| OsAffx.29778.1.S1_at | lectin-like receptor kinase, putative |
| OsAffx.29784.1.S1_at | heavy metal-associated domain containing protein |
| OsAffx.17626.1.S1_s_at | expressed protein |
| Os.17076.1.S1_at | cytochrome P450, putative, expressed |
| OsAffx.19409.1.S1_at | hypothetical protein |
| Os.54989.1.S1_at | ubiquitin-conjugating enzyme domain containing protein, expressed |
| OsAffx.29831.1.S1_s_at | expressed protein |
| Os.6976.2.A1_at | expressed protein |
| Os.53954.1.S1_at | plant protein of unknown function domain containing protein, expressed |
| OsAffx.6270.1.S1_at | pollen signalling protein with adenylyl cyclase activity, putative, expressed |
| Os.53458.1.S1_at | O-methyltransferase, putative, expressed |
| OsAffx.31208.1.S1_at | receptor kinase, putative |
| OsAffx.31220.1.S1_at | expressed protein |
| Os.34523.1.S1_at | HVA22, putative, expressed |
| Os.10185.1.S1_at | expressed protein |
| OsAffx.31250.1.S1_at | expressed protein |
| Os.10817.3.S1_x_at | auxin response factor, putative, expressed |
| Os.9774.1.S1_at | expressed protein |
| Os.9454.1.S1_at | expressed protein |
| OsAffx.7280.1.S1_at | expressed protein |

**Table S4.** *Cont.*

| Os.35907.1.S1_at | plant protein of unknown function domain containing protein, expressed |
| --- | --- |
| OsAffx.7287.1.S1_at | plant protein of unknown function domain containing protein |
| Os.52499.1.S1_at | stripe rust resistance protein Yr10, putative, expressed |
| OsAffx.19171.1.S1_at | protein kinase domain containing protein, expressed |
| Os.27573.1.S1_at | BSD domain-containing protein, putative, expressed |
| Os.8629.1.S1_x_at | LTPL12 - Protease inhibitor/seed storage/LTP family protein precursor, expressed |
| OsAffx.19473.1.S1_at | OsFBX438 - F-box domain containing protein, expressed |
| Os.21635.1.S1_at | major facilitator superfamily antiporter, putative, expressed |
| Os.7585.1.S1_at | transporter-related, putative, expressed |
| Os.21415.1.S1_at | TLD family protein, putative, expressed |
| Os.17909.1.S1_a_at | dehydrogenase E1 component domain containing protein, expressed |
| Os.46288.1.A1_at | thioredoxin, putative, expressed |
| Os.15763.1.S1_at | las1-like family protein, putative, expressed |
| Os.26801.1.A1_at | aspartic proteinase nepenthesin precursor, putative, expressed |
| Os.50368.1.S1_at | OsFBDUF66 - F-box and DUF domain containing protein, expressed |
| Os.52955.1.A1_at | expressed protein |
| OsAffx.7867.1.S1_at | conserved hypothetical protein |
| Os.8576.1.S1_at | 2-aminoethanethiol dioxygenase, putative, expressed |
| Os.27272.1.S1_at | deoxyhypusine hydroxylase, putative, expressed |
| Os.51071.1.A1_at | peptide transporter PTR2, putative, expressed |
| Os.52621.1.S1_x_at | peptide transporter PTR2, putative, expressed |
| Os.52529.1.S1_x_at | Unkown function or not predicted |
| Os.21788.1.S1_at | Unkown function or not predicted |
| Os.33341.2.S1_at | Unkown function or not predicted |
| Os.39985.1.A1_at | Unkown function or not predicted |
| Os.11986.3.S1_x_at | Unkown function or not predicted |
| Os.11986.3.S1_at | Unkown function or not predicted |
| Os.51807.1.S1_at | Unkown function or not predicted |
| Os.56133.1.S1_at | Unkown function or not predicted |
| Os.24115.1.A1_at | Unkown function or not predicted |
| Os.8863.1.S1_at | Unkown function or not predicted |
| Os.36350.1.A1_at | Unkown function or not predicted |
| Os.57563.1.S1_at | Unkown function or not predicted |
| Os.52581.1.S1_at | Unkown function or not predicted |
| Os.23112.1.A1_at | Unkown function or not predicted |
| OsAffx.29851.1.S1_at | Unkown function or not predicted |
| Os.51405.1.S1_at | Unkown function or not predicted |

**Table S4.** *Cont.*

| OsAffx.19154.1.S1_at | Unkown function or not predicted |
| --- | --- |
| Os.50762.1.S1_at | Unkown function or not predicted |
| Os.54454.1.S1_at | Unkown function or not predicted |
| Os.55261.1.S1_at | Unkown function or not predicted |
| Os.51767.1.S1_at | Unkown function or not predicted |
| OsAffx.12287.1.S1_s_at | Unkown function or not predicted |
| Os.14835.1.S1_x_at | Unkown function or not predicted |
| OsAffx.24724.1.S1_x_at | Unkown function or not predicted |
| Os.55114.1.S1_at | Unkown function or not predicted |
| Os.5769.1.S1_at | Unkown function or not predicted |
| Os.50522.1.S1_at | Unkown function or not predicted |
| Os.5823.1.S1_at | Unkown function or not predicted |
| Os.8195.1.S1_at | Unkown function or not predicted |

© 2013 by the authors; licensee MDPI, Basel, Switzerland. This article is an open access article distributed under the terms and conditions of the Creative Commons Attribution license (http://creativecommons.org/licenses/by/3.0/).
